# New Insights into Risk Genes and Their Candidates in Multiple Sclerosis

**DOI:** 10.3390/neurolint15010003

**Published:** 2022-12-29

**Authors:** Remina Shirai, Junji Yamauchi

**Affiliations:** Laboratory of Molecular Neurology, Tokyo University of Pharmacy and Life Sciences, 1432-1 Horinouchi, Hachioji 192-0392, Japan

**Keywords:** multiple sclerosis, oligodendrocyte, risk gene

## Abstract

Oligodendrocytes are central nervous system glial cells that wrap neuronal axons with their differentiated myelin membranes as biological insulators. There has recently been an emerging concept that multiple sclerosis could be triggered and promoted by various risk genes that appear likely to contribute to the degeneration of oligodendrocytes. Despite the known involvement of vitamin D, immunity, and inflammatory cytokines in disease progression, the common causes and key genetic mechanisms remain unknown. Herein, we focus on recently identified risk factors and risk genes in the background of multiple sclerosis and discuss their relationships.

## 1. Introduction

The myelin sheath is formed as a multilamellar membrane structure through the spiral wrapping of neuronal axons that act as insulators [1,2,3,4]. The transmission of each action potential on a limited membrane region is significantly promoted by the resulting saltatory conduction. Electrical signals are quickly derived to adjacent or distant neuronal cells and neuronal networks. If the myelin is damaged, however, fast signal transmission is not achieved, which causes defective neuronal function. This phenomenon is typically observed in demyelinating states. One well-known demyelinating disease is multiple sclerosis (MS) of the central nervous system (CNS). It is thought that MS is often caused by an abnormal autoimmune reaction in the CNS.

First defined by the National Multiple Sclerosis Society (in the United States) in 1996, MS is a chronic inflammatory disease that is characterized by demyelination mainly in the brain and, in turn, axonal degeneration [5]. The prevalence of MS is higher in Caucasians in the United States and Europe. The incidence rate is more than 100 patients per 100,000 population members in some areas of Northern Europe [6]. In Japan, the prevalence was estimated to be 1 to 5 patients per 100,000 population members, but this number has reportedly increased to 14 to 18 over a single decade [7]. The incidence of MS is increasing in both developed and developing countries [8]. The average age of onset of MS is middle-age and the disease is approximately twice as common in women than in men [9,10].

It is unclear why the number of MS patients has increased recently across countries and regions. There are various risks and possible reasons for the development of the disease, including smoking, vitamin D deficiency, obesity, and Epstein-Barr virus, which is a type of herpes virus [11]. MS also has genetic factors, as first-degree relatives and identical twins have a 25% chance of being affected [12]. The major histocompatibility complex (MHC) HLA-DRB1*15:01 allele was the first factor identified as a risk factor for MS [13]. Subsequent studies have shown that interleukin (IL) 2Rα and IL7R are also genetic factors [14].

MS symptoms likely depend on the tissues and regions where demyelination occurs. Some of the most common symptoms are optic neuritis and brainstem and spinal cord syndromes. Early clinical symptoms usually recover, but relapses are often followed by sequelae [15]. The onset is related to the location and size of the lesion, and even small lesions in the symptomatic zone are likely to cause symptoms, with magnetic resonance imaging showing typical “Dawson’s fingers” with periventricular lesions [16].

This narrative review will focus on the previously reported major risk factors of MS. We describe the disease and the possible therapeutic signaling pathways related to the risk factors as well as risk gene products in MS. We have selected the references using inclusion criteria that focus on reviews of MS and original papers. We have also included large-scale meta-analyses of original genetic studies.

## 2. MS and Environmental Factors (Vitamin D)

**Figure 1:**
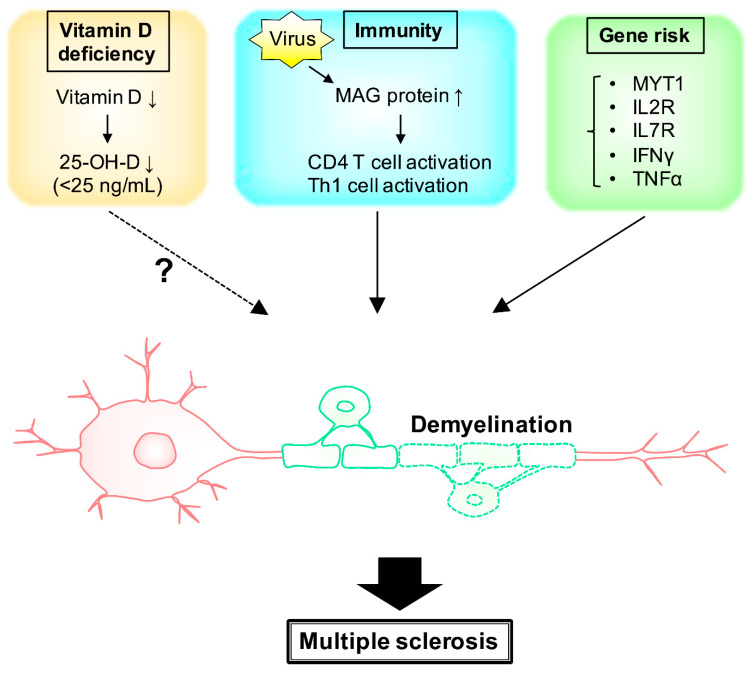
**Schematic diagram of some putative major factors associated with multiple sclerosis.** Deficiency of vitamin D results in demyelination. The levels of 25-OH-D, a metabolite from vitamin D, is one of the risks of MS. MAG proteins, as well as signaling molecules around immune cells, are also related to MS demyelination.

Epidemiological studies have shown that there are racial differences in developing MS, with a minimum prevalence at the equator and an increase with northern or southern latitudes [17]. Vitamin D is produced primarily by the action of ultraviolet B rays on the skin. This is supported by circumstantial evidence suggesting environmental factors of vitamin D deficiency due to a lack of sunlight as a predisposing factor for MS [18]. Indeed, vitamin D deficiency has been suggested as a possible cause of MS and/or contributor to the progression of MS, but it likely has limited pathological effects [19]. It has been reported that people with blood 25-OH-D levels of 40 ng/mL or higher have a 62% lower risk of MS than those with levels below 25 ng/mL [20], suggesting that having normal vitamin D levels reduces the risk of MS [9]. Serum 25-OH-D is a metabolite of vitamin D used to assess vitamin D levels in vivo (Figure 1) [20]. Adil et al. reported levels of vitamin D bioavailability and adipose tissue–secreted hormones such as adiponectin and leptin [21]. MS risk correlated with a genetic predisposition to the body mass index (BMI) but anti-correlated with the 25-OH-D level. Leptin and adiponectin have no effect on the increased risk of MS due to lowered vitamin D levels. Vitamin D supplementation modestly reverses the effect of obesity on MS [22]. In support of this study, Michaela et al. examined the association between 52 risk variants identified through genome-wide association studies (GWASs) and disease severity in MS and found that they were not associated with MS severity in terms of cohort, gender, age of onset, and HLA-DRB1*15:01 allele [21].

Scazzone et al. investigated the effects of vitamin D–related genes on MS susceptibility. Of the 12 vitamin D gene product pathways investigated, the most studied was the vitamin D receptor and the least studied were other vitamin D–related gene products. Scazzone et al. reported that it is not clear whether these mutations directly affect the risk of MS [23]. When vitamin D supplementation is used as a treatment, no statistically significant differences were found and its effectiveness could not be demonstrated [19,24]. Despite the lack of significant difference, vitamin D alters the transcriptome profile of macrophages and microglia. In addition, vitamin D activates T cells (Figure 1) [25]. MS risk and genetic abnormalities in vitamin D metabolism have been reported in several cases, with genetic abnormalities for CYP27B1 in the cytochrome P450 family gene products, which is a regulator of calcitriol synthesis, influencing MS risk [26].

Genetic polymorphisms in the gene-encoding molecules involved in vitamin D homeostasis are associated with vitamin D deficiency. However, Klotho, which is coded as a protein with vitamin D metabolism, has no genotypic frequencies that differ between MS patients and controls [27]. This finding means that the role of Klotho does not involve genetic susceptibility to MS.

Several studies have strengthened the candidacy of the environmental factors between vitamin D and the most major risk gene HLA-DRB1*15:01 allele. Vitamin D deficiency is also reported to be an important MS disease pathogenesis.

## 3. MS and Environmental Factors (Immunity)

CNS fibers are covered with myelin sheaths whose composition contains abundant lipids and proteins. In MS, demyelinating plaques are involved in an immune response triggered by T cells. Proteolipid protein (PLP), myelin basic protein (MBP), and myelin oligodendrocyte glycoprotein (MOG) proteins have been well-studied as self-antigens involved in demyelination in MS [28]. The autoimmune disease against MOG is called MOG antibody (MOG-IgG)-associated disease [29]. The clinical features are considered to reflect a unique disease with a different etiology from MS and optic neuritis, related to aquaporin-4 (AQP-4)-IgG [30,31].

Immune responses to myelin-associated glycoprotein (MAG) have been primarily implicated in the development of MS. Increased MAG-recognizing T and B cells in MS patients have been observed [32]. However, the MAG peptide itself did not elicit disease-specific T and B cell responses, suggesting that this is secondary to demyelination rather than an attack on MAG by immune responses [33]. In addition to MAG proteins, environmental factors such as viral infection trigger demyelination somewhat. Then, differentiation into CD4-positive T cells and Th1-type cells results in one of the key events in the early stages of MS (Figure 1) [34].

Most of the more than 100 mutations in MS reported to date are related to the human leukocyte antigen (HLA) and the immune system, supporting the idea that MS is an immune disease. However, these mutations account for only 25% of heritability, leading to the new concept of “phantom heritability.” Sawcer et al. proposed insufficient non-redundant unnecessary sufficient (INUS), which describes the plurality of causation when a mutation cannot be found [35].

Experimental autoimmune encephalomyelitis (EAE) is a typical mouse model of MS and has also been the basis for its etiology and therapeutic development with regard to induced CNS inflammation. A few researchers have put forth that the debate should not be focused on EAE, arguing that the phenotype is weak [36]. Microglia, macrophages, and dendritic cells, which are potent antigen-presenting cells, have been reported to be increased in EAE mice [37]. Microglia and macrophages are present in MS lesions, myelin proteins MBP, and PLP as well as the minor myelin proteins [38]. Faber et al. compared gene expression in opticospinal EAE (OSE) and MOG EAE models. They demonstrated a more extensive enrichment of human MS risk genes among transcripts differentially expressed in OSE than in MOG EAE [39].

When Li et al. analyzed the transcriptional profiling data in the human brain in MS, 133 known and unknown genes were identified [40]. They included genes encoding a number of extracellular matrixes, such as collagen, signal-triggering receptor, and molecules involved in immune-related pathways and phosphatidylinositol-3 kinase (PI3K)-Akt pathways. Among them, four major extracellular and transmembrane proteins, IL17A, IL2, CD44, and IGF1, and 16 extracellular proteins interacting with IL17A have been associated with MS pathogenesis. Additionally, Del-1, which is an interacting protein with IL17A that may be associated with MS progression and relapse, has been identified as a probable biomarker.

Regulatory T cell (Treg) alteration has also been implicated in the pathogenesis of MS. X-linked forkhead box P3 (FoxP3) plays a crucial role in the development and stability of Tregs. However, FoxP3 and vitamin D3 did not have any association with MS [41].

The humoral immune response to Epstein-Barr virus nuclear antigen 1 (EBNA-1)-specific immunoglobulin γ (IgG) titers in families with MS was determined as a result of investigating the role of specific genetic loci on the antiviral IgG titers. The EBNA-1 IgG gradient being the highest in MS patients and the lowest in biologically unrelated spouses indicates a genetic contribution to EBNA-1 IgG levels that is only partially explained by HLA-DRB1*15:01 carriership [42].

Although it was previously known that non-coding RNAs (ncRNAs) create transcription noise, they are also now believed to be regulators of immune responses. Dysregulation of ncRNAs is one of the underlying mechanisms of immune disorders such as MS. Several studies have reported the aberrant expression of ncRNAs in the sera or blood cells of MS patients [43,44,45]. The results of these studies propose different classes of ncRNAs (long non-coding RNAs, microRNAs, and circular RNAs) as diagnostic or predictive markers in MS [46].

Demyelinating plaques are related to the autoimmune response in MS. The production of inflammatory cytokines caused by the immune response, such as PLP, MBP, and MOG, revealed MS as a chronic inflammatory disease.

## 4. Gene Risk and Signaling Pathway

Environmental cues associated with the increased risk of developing MS have been established, and over 200 risk loci with moderate to subtle effects have been described. To dissect the influence of genetic predisposition and environmental factors, Florian et al. investigated the peripheral immune signatures of 61 monozygotic twin pairs discordant for MS. They revealed an inflammatory shift in a monocyte cluster of twins with MS, coupled with the emergence of a population of naive helper T cells that have a transient response IL2 as MS-related immune alterations [47]. The research on genetically identical (monozygotic) twins shows that the concordance rate for MS is approximately 30%. This indicates that genetic and environmental factors interact with MS. Baranzini et al. examined DNA methylation and gene expression across the genome in three monozygotic twins discordant for MS; however, there were no consistent differences in DNA sequence [48]. It is surprising that the environment strongly indicated epigenetic modifications to germline susceptibility based on studies of adoptees, half-siblings, and avuncular pairs. The fact that complete explanations for disease heritability were unachieved after whole-genome association studies warrants consideration of all the factors contributing to disease risk, such as genetic, epigenetic, and environmental factors [49].

As many as 200 single-nucleotide polymorphisms (SNPs) are associated with MS risk (Table 1) [50,51,52,53]. Gresle et al. analyzed MS risk expression quantitative trait loci associations for 129 distinct genes in MS patients [54]. They identified the MS risk SNPs, rs2256814 Myelin transcription factor 1 (MYT1) in CD4 cells and rs12087340 RF00136 in monocyte cells. IL7 receptor (IL7R) is a member of the type I cytokine receptor family and is a primary pleiotropic receptor in immune cells (Figure 1). Two GWASs of MS reported that three SNPs outside of the MHC region were associated with MS: rs6897032 within the IL7R gene and two SNPs (rs2104286 and rs12722489) in the IL2R gene [14,55]. Omraninava et al. revealed that the IL7RA gene rs6897932 SNP decreases MS susceptibility (Figure 1) [56]. Infection with the herpes virus and Mycoplasma pneumonia create grounds for MS. The T allele in the IFNγ gene (+874) and the genotypes of AA and AG at the TNFα gene (-308) at position−308 were considered potential risk factors for MS (Figure 1) [57]. Despite GWASs explaining that there are common SNPs associated with various diseases, known common variants only account for part of the estimated heritability of common complex diseases. Nadia et al. identified the rare functional variants analyzed within a large Italian MS multiplex family with five affected members [58]. Another recent study showed that up to 5% of MS inheritability may be accounted for by rare variations in the gene coding sequence, with four novel low genes driving MS risk independently of common variant signals [59]. Based on the research of a large cohort of Italian individuals, researchers identified three SNPs (rs4267364, rs8070463, rs67919208) that were involved in the regulation of TBK1 Binding Protein 1 (TBKBP1) and prioritized them as functionally relevant in MS [60]. Recent GWAS research in MS that has analyzed up to 47,000 MS patients and 68,000 healthy controls has determined more than 200 non-MHC genome-wide associations. The results show that immune cells, such as T cells, B cells, and monocytes, have susceptible gene specificity [61]. The International Multiple Sclerosis Consortium analyzed the large-scale GWAS data of 47,000 MS patients and 68,000 healthy controls and established a reference genetic map of MS. Their findings demonstrate the enrichment of MS genes in these brain-resident immune cells, suggesting that they may have a role in targeting an autoimmune process to the CNS, although MS is most likely initially triggered by a perturbation in peripheral immune responses [52].

**Figure 2:**
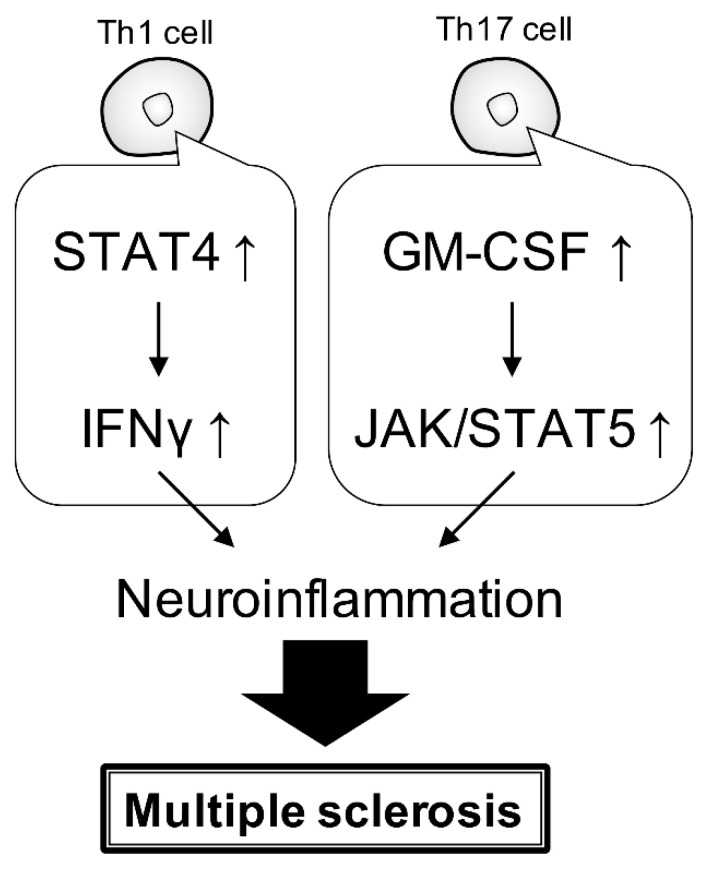
**JAK/STAT signaling pathway associated with MS.** Cytokines, through JAK/STAT signaling, especially in Th1 and Th17 cells, are putatively considered responsible for the progression of MS.

The Janus kinase and signal transducer and activator of the transcription (JAK/STAT) pathway is essential for both innate and acquired immunity. It has also been reported to be associated with several neuroinflammatory diseases (Figure 2) [62]. In EAE mice, Th1 cells produce interferon-gamma (IFNγ) via STAT4 and inflammatory macrophages, which promote macrophage activation. Similarly, Th17 produces granulocyte-macrophage colony-stimulating factor (GM-CSF) in the CNS and promotes macrophage polarization to inflammation via JAK/STAT5 (Figure 2) [63].

**Figure 3:**
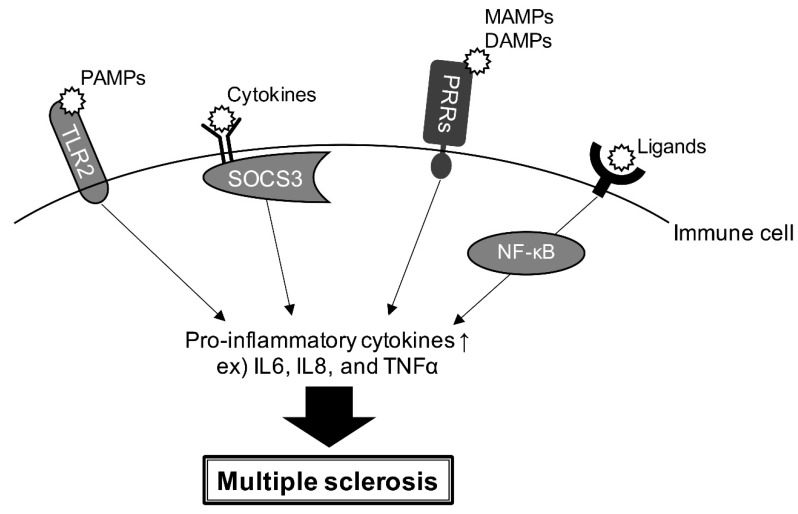
Interaction of some receptors with their cognate ligands induces the expression of pro-inflammatory cytokines in immune cells. Pathogen-associated molecular patterns interaction with TLRs, SOCS3 activation by cytokine receptors, microbe-associated molecular patterns or damage-associated molecular patterns binding to pattern recognition receptors, and/or activation through NF-κB are involved in the regulation of the expression of inflammatory cytokines, which are responsible for MS, in immune cells.

A comprehensive analysis of genes in the brain of MS patients has shown increased levels of immune cell populations and decreased ones of endothelial cells, Th1 cells, and Treg cells in MS lesions [64]. Toll-like receptors (TLRs) have a variety of roles, including axonal pathway formation and dorsoventral patterning in the CNS. TLR ligands, such as pathogen-associated molecular patterns (PAMPs), have been identified as T cell promoters in MS. In particular, TLR2 expression is high in MS lesions and TLR2 activation induces the expression of pro-inflammatory cytokines such as IL-6, IL-8, and TNF-α, which are implicated in exacerbated inflammation (Figure 3) [65]. The HLA signal in the Italian population maps to a glycoprotein involved in dendritic cell (DC) maturation, such as TNFSF14 gene encoding LIGHT. Miriam et al. reported that the TNFSF14 intronic SNP rs1077667 was the main MS-associated variant in the region. That means that the intronic variant rs1077667 alters the expression of TNFSF14 in DCs, which may play a role in MS pathogenesis [66]. A variant in TNHSH13B, encoding the cytokine and drug target B-cell activating factor (BAFF), was associated with upregulated humoral immunity through increased levels of soluble BAFF, B lymphocytes, and immunoglobulins in MS [67]. Leptin (LEP) and leptin receptor (LEPR) overexpression are related to MS activity and progression, and peroxisome proliferator-activated receptor gamma co-activator 1-alpha (PGC1A) is able to affect the reactive oxygen species production in the pathogenesis of MS. LEP rs7799039 and LEPR rs1137101 genetic variants modify the serum LEP levels and PGC1A rs8192678 alters the PGC1A activity. Ivana et al. revealed that the PGC1A rs8192678 minor allele had an increased risk for the occurrence of MS, and LEP rs7799039 affected the LEP gene expression in relapsing-remitting patients [68].

Furthermore, in relapsing MS, reduced suppression of cytokine signaling-3 (SOCS3) expression in the CNS and immune cells may induce LEP-mediated overexpression of pro-inflammatory cytokines (Figure 3) [69]. Pattern recognition receptors, which are triggered by both microbe-associated molecular patterns and damage-associated molecular patterns, have been reported to regulate innate immune responses in MS and an EAE model. Pattern recognition receptor signaling promotes inflammatory-producing cytokine production in CNS autoimmune diseases (Figure 3) [70]. NF-κB is involved in a wide range of vital processes, including inflammation, cell proliferation, and differentiation. Abnormal NF-κB activation has been reported to be closely associated with the development of MS and EAE [71].

**Figure 4:**
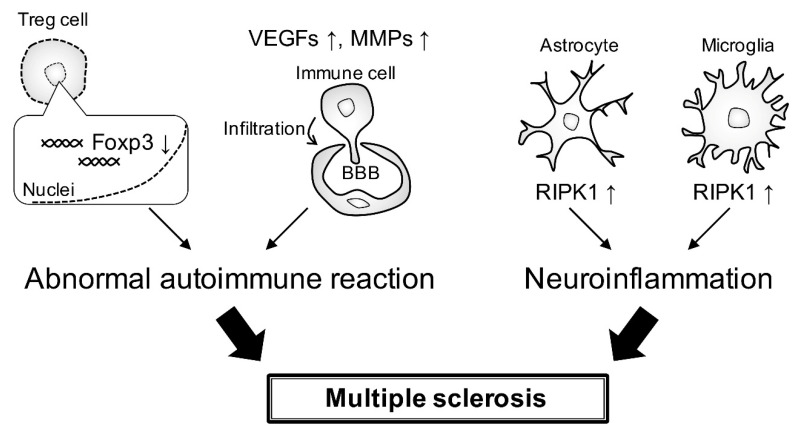
**Abnormal autoimmune reaction and neuroinflammation in MS.** Altered Foxp3 expression in Treg cells induces an abnormal autoimmune reaction. Expression levels of vascular endothelial growth factors and matrix metallopeptidases are increased, probably disrupting the blood–brain barrier. This disruption allows immune cells to infiltrate. Phosphorylation of RIPK1 in astrocytes and microglia is involved in the promotion of the neuroinflammatory program.

In MS, the altered Foxp3-E2 variant-associated inhibitory activity of Treg cells is associated with defective signaling via IL-2 and glycolysis, which modulates Treg cell induction and function in autoimmunity [72]. The expression of vascular endothelial growth factors and matrix metallopeptidases involved in angiogenesis is increased in MS. These genes are also involved in basement membrane degradation and blood–brain barrier disruption, which allows immune cells to infiltrate the CNS in EAE and MS (Figure 4) [73]. Programmed cell death 1 (PD-1) is known as an immune checkpoint that is associated with several autoimmune diseases. Research on the frequency of PD-1 genotypes and alleles in MS patients shows that PD-1 gene polymorphisms may be associated with MS [74]. Phosphorylation of receptor-interacting protein kinase 1 (RIPK1) in astrocytes and microglia triggers a detrimental neuroinflammatory program that contributes to the neurodegenerative environment in MS (Figure 4) [75].

Risk genes have been well studied by meta-analyses and many SNPs have been identified. MYT1, IL2R, IL7R, IFNγ, and TNFα, among others, are considered to be the major risk genes in MS. The related major signaling in MS is the JAK/STAT pathway.

## 5. Conclusions and Perspective

We have examined and discussed the genetic risks in the background of MS. The major risks include (1) the genes related to vitamin D deficiency, (2) the genes involved in the immune response, and (3) the genes responsible for inflammatory cytokines and the related signaling molecules. Nucleotide sequence analyses with advancing technologies have clarified that there are an increasing number of other possible categories of risk genes besides these in MS. In the future, molecules related to these risk gene products may be promising therapeutic target candidates.

## Figures and Tables

**Table 1:** **The risk allele and its possible role for the 200 autosomal non-MHC genome-wide effects.** This list shows the 200 SNP regions and the possible roles of probable genes associated with MS risks, as identified by SNP analyses [50,51,52,53].

SNP Region	Gene	Protein	Possible Role of Nearest Gene
rs6742	rtel1	RTEL1	DNA helicase
rs32658	fam170a	FAM170A	DNA binding activator
rs137955	rpl3	RPL3	ribosomal protein
rs140522	hdac10	HDAC10	deacetylase
rs198398	mtor	MTOR	rapamycin kinase
rs244656	ppp2ca	PPP2CA	catalytic subunit of protein phosphatase
rs249677	arhgap26	ARHGAP26	GTPase activating protein
rs354033	znf862	ZNF862	zinc finger protein
rs405343	axin1	AXIN1	cytoplasmic protein
rs438613	eomes	EOMES	DNA binding domain
rs483180	notch2	NOTCH2	notch receptor
rs531612	rela	RELA	proto-oncogene transcription factor
rs631204	tnfaip3	TNFAIP3	cytokine
rs701006	arhgap9	ARHGAP9	GTPase activating protein
rs719316	atxn1	ATXN1	DNA binding protein
rs735542	myc	MYC	proto-oncogene transcription factor
rs760517	lgals1	LGALS1	galactoside binding protein
rs802730	ptprk	PTPRK	protein tyrosine phosphatase receptor
rs883871	rara	RARA	retinoic acid receptor
rs962052	rnd3	RND3	Rho family GTPase
rs983494	cd48	CD48	immune response regulator
rs1014486	il12a	IL12A	cytokine
rs1026916	hoxa13	HOXA13	homeobox
rs1076928	pim1	PIM1	proto-oncogene kinase
rs1077667	c3	C3	complement component
rs1087056	znf438	ZNF438	zinc finger protein
rs1112718	ide	IDE	insulin enzyme
rs1177228	commd1	COMMD1	copper metabolism
rs1250551	zmiz1	ZMIZ1	zinc finger protein
rs1323292	rgs1	RGS1	G Protein Signaling
rs1365120	traf6	TRAF6	adaptor protein
rs1399180	gata3	GATA3	transcription factor
rs1415069	bcar3	BCAR3	anti-estrogen resistance protein
rs1465697	atf5	ATF5	transcription factor
rs1738074	synj2	SYNJ2	inositol polyphosphate 5-phosphatase
rs1800693	cd9	CD9	immune response regulator
rs2084007	ppp2ca	PPP2CA	catalytic subunit of protein phosphatase
rs2150879	rps6kb1	RPS6KB1	ribosomal protein
rs2248137	znf217	ZNF217	zinc finger protein
rs2269434	celf1	CELF1	alternative splicing
rs2286974	litaf	LITAF	cytokine
rs2289746	alcam	ALCAM	immunoglobulin receptor
rs2317231	cd1e	CD1E	immune response regulator
rs2327586	sgk1	SGK1	serine/threonine kinase
rs2331964	cd86	CD86	immune response regulator
rs2364485	cd9	CD9	immune response regulator
rs2469434	cd226	CD226	immune response regulator
rs2546890	il12b	IL12B	cytokine
rs2585447	znf217	ZNF217	zinc finger protein
rs2590438	bcl6	BCL6	immune signaling receptor
rs2705616	mapk10	MAPK10	MAPK
rs2726479	cxxc4	CXXC4	zinc finger protein
rs2836438	ets2	ETS2	transcription factor
rs2986736	camta1	CAMTA1	transcription activator
rs3184504	arpc3	ARPC3	cell polymerization
rs3737798	cd48	CD48	immune response regulator
rs3809627	mapk3	MAPK3	MAPK
rs3923387	plec	PLEC	cytoskeleton
rs4262739	ets1	ETS1	proto-oncogene transcription factor
rs4325907	rpl24	RPL24	ribosomal protein
rs4409785	maml2	MAML2	cytoplasmic protein
rs4728142	smo	SMO	G protein-coupled receptor
rs4796224	acaca	ACACA	acetyl-CoA carboxylase
rs4808760	ifi30	IFI30	lysosomal thiol reductase
rs4812772	mybl2	MYBL2	proto-oncogene transcription factor
rs4820955	lif	LIF	cytokine
rs4896153	bclaf1	BCLAF1	BCL transcription factor
rs4939490	fads1	FADS1	fatty acid desaturase
rs4940730	malt1	MALT1	caspase-like protease
rs5756405	rac2	RAC2	GTP binding protein
rs6020055	cebpb	CEBPB	transcriptional activator protein
rs6032662	cd40	CD40	immune response regulator
rs6072343	plcg1	PLCG1	phospholipase
rs6427540	cd48	CD48	immune response regulator
rs6496663	iqgap1	IQGAP1	GTPase activating protein
rs6533052	nfkb1	NFKB1	cytokine
rs6564681	maf	MAF	proto-oncogene kinase
rs6589706	kmt2a	KMT2A	Lysine Methyltransferase
rs6589939	clmp	CLMP	transmembrane protein
rs6670198	prdm16	PRDM16	zinc finger protein
rs6672420	runx3	RUNX3	transcription factor
rs6738544	stat1	STAT1	transcription activator
rs6789653	zbtb38	ZBTB38	zinc finger protein
rs6837324	tec	TEC	tyrosine kinase
rs6911131	hivep2	HIVEP2	zinc finger protein
rs6990534	myc	MYC	proto-oncogene transcription factor
rs7222450	crhr1	CRHR1	G-protein coupled receptor
rs7260482	apoe	APOE	apoprotein
rs7731626	map3k1	MAP3K1	MAPK kinase
rs7855251	anp32b	ANP32B	RNA polymerase binding protein
rs7975763	mphosph9	MPHOSPH9	M phase phosphoprotein
rs7977720	olr1	OLR1	low density lipoprotein receptor
rs8062446	nlrc5	NLRC5	cytokine receptor
rs9308424	batf3	BATF3	basic leucine zipper protein
rs9568402	rnaseh2b	RNASEH2B	ribonuclease
rs9591325	rnaseh2b	RNASEH2B	ribonuclease
rs9610458	ube2l3	UBE2L3	ubiquitin conjugating enzyme
rs9808753	ifnar2	IFNAR2	interferon receptor
rs9843355	cd80	CD80	immune response regulator
rs9863496	satb1	SATB1	matrix protein
rs9878602	rybp	RYBP	DNA binding protein
rs9900529	grb2	GRB2	growth factor receptor
rs9909593	rara	RARA	retinoic acid receptor
rs9955954	malt1	MALT1	caspase-like protein
rs9992763	rpl34	RPL34	ribosomal protein
rs10063294	slc1a3	EAA1	transporter
rs10191360	cxcr4	CXCR4	chemokine receptor
rs10230723	ikzf1	IKAROS	DNA binding protein
rs10245867	hoxa13	HOXA13	homeobox
rs10271373	tbxas1	TBXAS1	lipid synthase
rs10801908	atp1a1	ATP1A1	transporting subunit
rs10936182	il12a	IL12A	cytokine
rs10936602	mecom	MDS1 And EVI1 Complex Locus	zinc finger protein
rs10951042	mad1l1	MAD1	cell cycle controller
rs10951154	hoxa4	HOXA4	homeobox
rs11079784	npepps	NPEPPS	peptidase
rs11083862	c5ar1	C5AR1	complement component receptor
rs11125803	adcy3	ADCY3	adenylate cyclase
rs11161550	bcl10	BCL10	immune signaling receptor
rs11231749	esrra	ESRRA	estrogen related receptor
rs11256593	pfkfb3	PFKFB3	phosphofructo kinase
rs11578655	extl2	EXTL2	glycosyltransferase
rs11749040	dab2	DAB2	adaptor protein
rs11809700	rpl5	RPL5	ribosomal protein
rs11852059	ptger2	PTGER2	prostaglandin receptor
rs11899404	lpin1	LPIN1	lipid phosphohydrolase
rs11919880	cnot10	CNOT10	transcription complex
rs12133753	cdc7	CDC7	cell cycle kinase
rs12147246	rcor1	RCOR1	transcription factor
rs12211604	rreb1	RREB1	binding protein
rs12365699	kmt2a	KMT2A	methyltransferase
rs12434551	zfp36l1	ZFP36L1	zinc finger protein
rs12478539	zfp36l2	ZFP36L2	zinc finger protein
rs12588969	rcor1	RCOR1	chromatin binding
rs12609500	tyk2	TYK2	tyrosine kinase
rs12614091	cd28	CD28	immune response regulator
rs12622670	aplf	APLF	component of the cellular response
rs12722559	pfkfb3	PFKFB3	glycolysis-related biphosphatase
rs12832171	cd9	CD9	immune response regulator
rs12925972	maf	MAF	proto-oncogene kinase
rs12971909	map2k2	MAP2K2	MAPK kinase
rs13066789	bcl6	BCL6	immune signaling receptor
rs13136820	uchl1	UCHL1	ubiquitin hydrolase
rs13327021	eomes	EOMES	DNA binding domain
rs13385171	sertad2	SERTAD2	transcription activator
rs13414105	alk	ALK	tyrosine kinase
rs17051321	qrfpr	QRFPR	pyroglutamylated receptor
rs17724508	maf	MAF	proto-oncogene kinase
rs17741873	camk2g	CAMK2G	CAM kinase
rs17780048	tnfaip3	TNFAIP3	cytokine
rs28703878	pkia	PKIA	protein kinase inhibitor
rs28834106	dnm2	DNM2	GTP binding protein
rs34026809	kmt2a	KMT2A	methyltransferase
rs34536443	tyk2	TYK2	tyrosine kinase
rs34681760	adcy2	ADCY2	adenylate cyclase
rs34695601	fos	FOS	proto-oncogene transcription factor
rs34723276	extl2	EXTL2	glycosyltransferase
rs34947566	litaf	LITAF	cytokine
rs35218683	deaf1	DEAF1	zinc finger protein
rs35486093	bcl10	BCL10	adaptor protein
rs35540610	sp110	SP110	nuclear body protein
rs35703946	irf8	IRF8	cytokine
rs55858457	mad1l1	MAD1L1	cell cycle controller
rs56095240	maml2	MAML2	transcriptional activator
rs57116599	il1b	IL1B	cytokine
rs58166386	rasal3	RASAL3	Ras GTPase activating protein
rs58394161	rpl5	RPL5	ribosomal protein
rs59655222	znf281	ZNF281	zinc finger protein
rs60600003	elmo1	ELMO1	adaptor protein
rs61708525	plxnc1	PLXNC1	transmembrane receptor
rs61863928	egr2	EGR2	transcription factor
rs61884005	arntl	ARNTL	transcriptional activator
rs62013236	acsbg1	ACSBG1	acyl-CoA synthetase
rs62420820	tnfaip3	TNFAIP3	cytokine
rs67111717	nsd1	NSD1	transcriptional regulator
rs67934705	rpl11	RPL11	ribosomal protein
rs71329256	cd86	CD86	immune response regulator
rs72922276	pde4b	PDE4B	phosphodiesterase
rs72928038	rragd	RRAGD	Ras related GTPase binding protein
rs72989863	march1	MARCH1	ubiquitin protein ligase
rs73414214	pik3cg	PIK3CG	Phosphoinositide 3-kinase
chr1:154983036	arhgef2	ARHGEF2	Rho/Rac guanine nucleotide exchanger
chr1:32738415	hdac1	HDAC1	histone deacetylase
chr2:112492986	anapc1	ANAPC1	anaphase-promoting complex
chr3:100848597	rpl24	RPL24	ribosomal protein
chr3:112693983	cd200	CD200	immune response regulator
chr3:121783015	cd86	CD86	immune response regulator
chr5:40429250	dab2	DAB2	DAB adaptor protein
chr6:119215402	mcm9	MCM9	ATP hydrolysis activity
chr6:130348257	arhgap18	ARHGAP18	Ras GTPase activating protein
chr6:14691215	jarid2	JARID2	transcriptional repressor
chr7:50328339	ikzf1	IKZF1	zinc finger protein
chr8:129177769	myc	MYC	proto-oncogene transcription factor
chr8:95851818	rad54b	RAD54B	DEAD-like helicase
chr11:118783424	kmt2a	KMT2A	lysine methyltransferase
chr11:14868316	pde3b	PDE3B	phosphodiesterase
chr13:100026952	dock9	DOCK9	Cdc42 guanine nucleotide exchanger
chr14:88523488	kcnk10	KCNK10	potassium channel protein
chr16:11213951	litaf	LITAF	cytokine
chr16:11353879	litaf	LITAF	cytokine

## Data Availability

Not applicable.
